# Evidence of multiple intraspecific transmission routes for *Leptospira* acquisition in Norway rats (*Rattus norvegicus*)

**DOI:** 10.1017/S0950268817002539

**Published:** 2017-12

**Authors:** A. MINTER, P. J. DIGGLE, F. COSTA, J. CHILDS, A. I. KO, M. BEGON

**Affiliations:** 1 Institute of Integrative Biology, The University of Liverpool, Liverpool, UK; 2 CHICAS, Lancaster University Medical School, Lancaster University, Lancaster, UK; 3 Instituto de Saúde Coletiva, Universidade Federal da Bahia, Salvador, Bahia, Brazil; 4 Centro de Pesquisas Gonçalo Moniz, Fundação Oswaldo Cruz, Ministério da Sáude, Salvador, Bahia, Brazil; 5 Department of Epidemiology of Microbial Diseases, Yale School of Public Health, New Haven, Connecticut, USA

**Keywords:** Age-prevalence, intraspecific transmission, leptospirosis, Norway rats

## Abstract

Infectious diseases frequently have multiple potential routes of intraspecific transmission of pathogens within wildlife and other populations. For pathogens causing zoonotic diseases, knowing whether these transmission routes occur in the wild and their relative importance, is critical for understanding maintenance, improving control measures and ultimately preventing human disease. The Norway rat (*Rattus norvegicus*) is the primary reservoir of leptospirosis in the urban slums of Salvador, Brazil. There is biological evidence for potentially three different transmission routes of leptospire infection occurring in the rodent population. Using newly obtained prevalence data from rodents trapped at an urban slum field site, we present changes in cumulative risk of infection in relation to age-dependent transmission routes to infer which intra-specific transmission routes occur in the wild. We found that a significant proportion of animals leave the nest with infection and that the risk of infection increases throughout the lifetime of Norway rats. We did not observe a significant effect of sexual maturity on the risk of infection. In conclusion, our results suggest that vertical and environmental transmission of leptospirosis both occur in wild populations of Norway rats.

## INTRODUCTION

There are often multiple potential routes of intraspecific transmission of pathogens within wildlife and other populations. Elaborating the various intra- and inter-specific routes of transmission and their relative importance are essential for understanding the dynamics of the agent, with implications for interventions to reduce infection prevalence in the reservoir host and to control or prevent subsequent human disease [1]. Obtaining evidence of transmission routes by experimental infection in a laboratory setting is difficult and often does not represent transmission as it would occur in the wild. Inferring the relative importance of different potential transmission routes from field data may, therefore, be of both fundamental and practical interest. However, inferring routes of transmission from statistical associations is not straightforward. There is a need to consider multiple statistical models with different underlying assumptions to better understand associations between risk and reality.

Leptospirosis is a zoonosis caused by pathogenic bacteria of the genus *Leptospira* [2]. Many mammals serve as reservoir hosts, becoming chronically infected within their kidneys and shedding infectious leptospires in urine. Humans are incidentally infected and there are very few cases of human-to-human transmission [3, 4]. The main routes of human infection are through contact with environmental sources contaminated with animal urine or through direct contact with animal reservoirs [2]. Available vaccines to prevent human leptospirosis are often not effective [5] as they do not protect against all *Leptospira* serovars, do not induce long-lasting immunity and have side effects [6]. Control of transmission within and from the reservoir host may, therefore, be critical for disease control.

Urban slum dwellers, who account for one-third of people living in urban settings, are at increased risk of water borne and zoonotic infectious disease, as a result of substandard housing and the lack of sanitary services [7]. Salvador, a coastal city in north-east Brazil, has experienced a recent human population increase, leading to the creation and expansion of urban slums [8]. Salvador, Brazil registers annual outbreaks of leptospirosis [8] where annual flooding events, associated with the rainy season, wash contaminated soil and water into areas of potential human use. In Pau da Lima, an urban slum in Salvador, several studies have demonstrated an increased risk of *Leptospira* transmission associated with residence regions prone to flooding, open sewers, proximity to accumulated refuse and sightings of rats at the home [9, 10]. The incidence of leptospirosis in the slums of Salvador is high. A recent community-based cross-sectional survey of 3171 slum residents found an overall prevalence of Leptospira antibodies of 15·4% [9].

Residents in the slums live in close proximity to the primary animal reservoir, the Norway rat (*Rattus norvegicus*) [8, 11, 12] and environmental reservoir contaminated with leptospires shed in rat urine. Isolates of leptospires infecting Norway rats in Salvador have been repeatedly identifies as *Leptospira interrogans* serovar Copenhagenii (serogroup Icterohaemorrhagiae) [13]. Paired results between qPCR of urine or kidney samples were previously shown to be 100% concordant [13]. The prevalence of *Leptospira* kidney carriage among rats in Salvador ranges between 60% and 80% and age-stratified rates of shedding are known [12, 13], but we do not currently understand the pathways of intra-specific transmission of leptospires in the rat reservoir. There are multiple potential routes: (1) vertical and pseudo-vertical transmission, where rats acquire infection in utero or acquire infection via suckling from infected dams (herein, for practical reasons, we make no attempt to distinguish these potential routes); (2) direct transmission, either by sexual contact, direct contact with urine or by some other direct mechanism such as bites; and (3) infection from exposure to environmental sources contaminated with bacteria. There is evidence that vertical and sexual transmission is biologically feasible, namely the presence of leptospires in the mammary gland, milk and semen of rats ([14] and Lin Zhan, unpublished observations) and high concentrations of leptospires are shed in the urine, so transmission through contact with the contaminated environment can be assumed to occur [13], but the epidemiological significance of the transmission routes is unknown.

We can gain insight into the contribution of various transmission routes by examining changes in the prevalence of leptospiral carriage related to increasing age and different life stages, such as sexual maturity. When rats are born, they are initially confined to the nest [15]. Once weaned, they leave the nest and begin to roam freely as sub-adults, eventually becoming sexually mature [15]. In urban settings, the time to sexual maturity is weight dependent and so animals can become sexually mature over a wider range of ages. Vertical and pseudo-vertical transmission, in addition to exposure within the burrow nest, must occur prior to weaning. Together these determine the proportion of animals infected once they first appear in the free-roaming population. Once rats leave the natal burrow they are exposed to environmental contamination and (non-sexual) direct transmission and after reaching sexual maturity they have the additional risk of direct transmission during sexual contact. For leptospirosis, wounding has been found to be associated in Norway rats with a higher *Leptospira* load in the urine and kidney [13, 16]. The level of wounding is also a risk factor for Hantavirus infection in wild rats [17], for which the primary route of infection is direct (via biting), but the presence of leptospires in saliva has not been tested for [13].

We wish to answer the question of which of the hypothesised transmission routes are biologically significant. Given the high level of prevalence, most rodents will become infected at some point in their lifetime. Identifying risk factors for being infected at the time of capture would not take into account the fact that rodents uninfected at time of capture are susceptible to future infection. By setting the problem in a survival framework we are better able to describe the relative importance of the multiple intra-specific transmission routes throughout the life-course and hence to indicate which sub-populations of rodents should be targeted for control. Previous studies of wildlife disease have used age-prevalence data to infer evidence of transmission routes based on the force of infection (FOI), also known as the hazard of infection. Our approach differs from previous studies which made *a priori* assumptions about how the risk of infection changes over time [18–20]. The FOI is ‘the per capita rate at which susceptible hosts acquire infection’ [21] and can be represented algebraically based on a mathematical framework or, in the case of data analysis, modelled as a survival distribution [22]. In our application, we employ a FOI approach using a flexible survival distribution with demographic covariates to model the hazard of infection.

Here, therefore, we take two complementary approaches. First, we identify risk factors for infection from demographic factors (sex, age/mass and external indications of sexual maturity) and the presence of bites or healing wounds. Second, we use a survival analysis to estimate the changing risk of infection as the rats age and seek evidence for differential risk among different sub-populations of rats. Thus, we present an extension to the practice of analysing age-prevalence data by considering the changes in cumulative risk of infection based on demographic variables related to age-dependent transmission routes.

## MATERIALS AND METHODS

### Study site

The study was carried out in the Pau da Lima neighbourhood, an urban slum community comprised of three valleys in Salvador. Salvador is the third largest city in Brazil, with almost two-thirds of its residents living in slums [7]. Human cases of leptospirosis occur annually, with a higher incidence in the rainy season [8].

### Data collection

Demographic information (sex, weight, body length, reproductive status (scrotal testes for males and perforate vagina, enlarged breasts or evidence of lactation for females), pregnancy status for females) and the presence and severity of wounds/scars were recorded from external examinations of Norway rats trapped over five collection periods (June–July 2012, May–August 2013, October–December 2013, March–August 2014, September–December 2014). For full details see [23]. Wounding grade, previously identified as a risk factor for leptospiral infection among Norway rats, was recorded on a five-point categorical scale 0 (absent), 1 (very light), 2 (light), 3 (moderate) and 5 (severe) following [24].

### Statistical analyses

#### Ageing field animals

Body weight has been used as a surrogate variable for estimating the age of Norway rats in most field studies [25, 26] but the weight is non-linearly related to age and so may be misleading in analyses of transmission patterns [15]. To overcome this limitation, we impute the age of rats from their weight using the von Bertalanffy equation for growth.

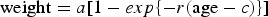

where *a* is the asymptote, *r* is the constant growth rate and *c* is the age at which maximum growth occurs [27]. We generated a standardised von Bertalanffy curve for wild Norway rats using weight and age data obtained from captive colonies kept in large outside enclosures [15]. In justification of this, the range of weights of male and female rats from Salvador was comparable with those obtained by [15] and weight distributions do not vary significantly by month or season of sampling, although distinct wet and dry seasons occur [23]. The fitted von Bertalanffy curve using the data from [15] had asymptote *a* = 562 days and estimated values for growth rate *r* = 0·01337 (grams per day) and point of inflection *c* = 23 days. All statistical analyses were performed in R [28]. Both male and female rats caught in the field had a similar range of weights, so we converted their weights to ages using one growth curve.

An additional complication to ageing female rats by weight is the potential for age misclassification associated with pregnancy. To address this concern, prior to imputing age among female rats we compared weights between visibly pregnant rats (*N* = 68 animals) and non-pregnant mature females (perforate vagina, placental scars indicating past pregnancy, or lactating, but not pregnant; *N* = 72 animals). The difference in the weights of pregnant and non-pregnant females was not significant (two sample *t*-test, mean difference =20·81, *t* = −1·41, df = 132·22, *P* = 0·159). This lack of effect may be due to an averaging out of the weights of animals at different stages of pregnancy or and inflation of error variance due to using the unmeasured age covariate. We nonetheless took the conservative step of adjusting the weights of pregnant females by the estimated mean difference as an average measure of pregnancy.

#### Prevalence analysis

Infection was modelled as a binary response variable. Infection status was determined by qPCR analysis of urine or kidney samples for a gene specific to pathogenic leptospires (*Lip32*), as described in detail elsewhere [13]. For 3·3% of the animals, urine could not be collected and infection was determined by the presence of leptospires in kidney qPCR.

There were 486 animals for which the presence of kidney carriage was established and complete records of all explanatory variables recorded: age, sex (male/female), weight, sexual maturity and level of wounding. The prevalence of leptospiral carriage was independent of collection time (*χ*^2^ = 6·02, degrees of freedom = 4, *P* = 0·20) and so this was not included as an explanatory variable in the model selection process.

Generalised linear models (GLMs) of leptospiral infection were fitted, the bias reduction method developed by [29, 30] was used as there was complete or quasi-complete separation present during the GLM selection. For ease of statistical computation, we collapsed the level of wounding [24] into three grades: 0 (absent), 1 (very light and light combined) and 2 (moderate and severe combined). Age was adjusted so that the intercept was at our lowest recorded imputed age (27 days). A male was classified as sexually mature by the presence of scrotal testes and a female by pregnancy, lactation or presence of placental scars. A GLM was fitted with adjusted age, sex, sexual maturity, wounds and all possible two-way interactions between those covariates. We chose to use a backward elimination approach with a 5% significance threshold to obtain the final prevalence model. The final model was then used to identify risk factors for acquiring infection and to estimate the risk of infection for rats at different developmental stages and with different classes of wounding.

#### Survival analysis

Our analysis of risk-factors for infection does not differentiate between recent and more long-standing infections. However, carriage in the urine and/or kidney suggests that exposure occurred at least 10 days prior to detection [31], so rats were infected at an unknown time prior to their capture. Additionally, uninfected animals at capture would have been at risk of subsequent infection at a later date. If *T* is the time of first infection, *t*_*i*_ is the observed time (age at capture) and *Y*_*i*_ is coded 0 and 1 to denote absence or presence of infection at capture, respectively, it follows that

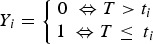


In other words, positive animals were infected at the age of capture or before and negative animals may be infected in the future. The probability of not yet being infected (according to being urine or kidney positive) at age *t* is the cumulative distribution function of *T*, which we model as a Weibull distribution, where *F*(*t*_*i*_) = 1 − exp(−(*t*_*i*_/*ϕ*)^*κ*^) with scale parameter *ϕ* and shape parameter *κ*. To investigate the effect of explanatory variables *x*_*i*_ on risk of infection, we specify a log-linear model for the scale parameter, log(*ϕ*) = *α* + *βx*_*i*_ Using the fact that *P*(*Y*_*i*_ = 0) = 1 − *F*(*t*_*i*_) we can use the binary data *Y*_*i*_ to estimate the parmeters of the survival distribution (for details, see Supplementary Materials).

To investigate the effect of multiple variables on the scale parameter (*ϕ*) and whether risk varied by age, we fitted a model with sex, maturity status and a binary wounding variable (absent/present) as factors and then tested for significant interactions between the variables. The shape parameter *κ* determines how the risk of infection changes over a rat's lifetime. For *κ* < 1 and *κ* > 1 the risk of infection decreases and increases, respectively, with age, whilst if *κ* = 1 the risk of infection is constant.

## RESULTS

### Ageing field animals

**Fig. 1.**
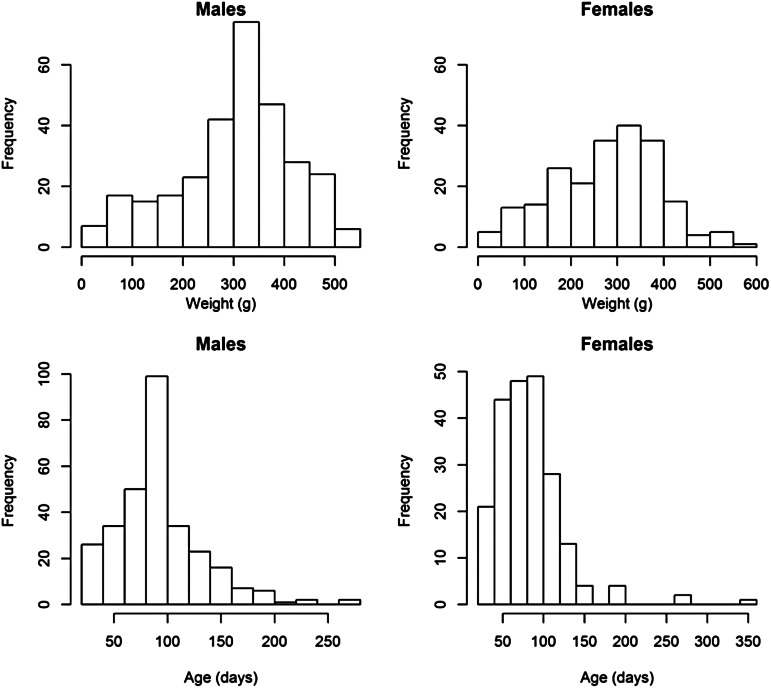
Histograms of the weights and estimated ages of male and female animals from the field.

Weight and age distributions are shown in Figure 1. Although males (mean weight 309·24) were on average heavier than females (mean = 281·23) (two sample *t*-test, *t* = 2·76, df = 460·7, *P* < 0·01), the weight distributions of males and females had similar ranges and shapes, which resulted in similar distributions of estimated ages (Fig. 1). Most animals had an estimated age of less than 100 days; few animals were over 200-days-old (10 in total).

### Prevalence analysis

**Fig. 2.**
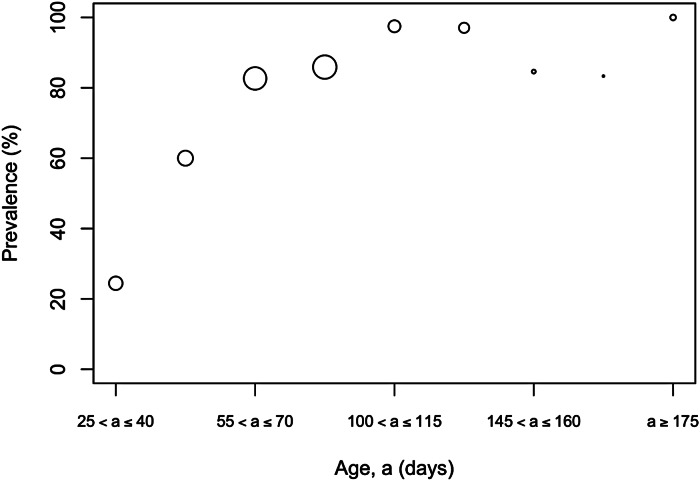
Observed prevalence against age for different age bins. Larger circles indicate a larger sample size for the age bin, the maximum sample size was *N* = 89 for ages (*a*) in the bin 90 ⩽ *a* < 105 days.

The overall prevalence of leptospiral carriage in the population was 80% (95% confidence interval: 0·76–0·84, *N* = 486). The age-prevalence profile is shown in Figure 2. All animals over the age of 175 days were infected (*N* = 19). Prevalence was independent of collection time (*χ*^2^ = 6·02, degrees of freedom = 4, *P* = 0·20) and so was not included as an explanatory variable in the model selection process (Table 1).

**Table 1.** Counts of animals negative and positive (%) for infection, sex, sexual status and wounding grade by collection time

	Collection time					
	June–July 2012	May–August 2013	October–December 2013	Mar–August 2014	September–December 2014	
Season	Rainy	Rainy	Dry	Rainy	Dry	Total
Infection						
Negative	7 (13)	25 (16)	28 (26)	22 (22)	14 (19)	96 (20)
Positive	46 (87)	129 (84)	78 (74)	77 (78)	60 (81)	390 (80)
Sex						
Male	28 (53)	93 (60)	62 (58)	56 (57)	44 (59)	283 (58)
Female	25 (47)	61 (40)	44 (42)	43 (43)	30 (41)	203 (42)
Sexual status						
Immature	8 (15)	32 (21)	25 (24)	20 (20)	7 (9)	92 (19)
Mature	45 (85)	122 (79)	81 (76)	79 (80)	67 (91)	394 (81)
Wounding						
Absent	26 (49)	84 (55)	56 (53)	39 (39)	13 (18)	218 (45)
Very light and light	12 (23)	36 (23)	24 (23)	47 (47)	48 (65)	167 (34)
Moderate and severe	15 (28)	34 (22)	26 (25)	13 (13)	13 (18)	101 (21)

The final GLM of leptospiral infection among rats included age, wounding, sexual maturity and an interaction between wounding and age. Risk of being infected increased with age, level of wounding and being sexually mature (Table 2), but the risk of infection no longer increased for older animals when they had higher levels of wounding.

**Table 2.** Summary of final generalised linear model (GLM) prevalence model fit (AIC = 376)

	Estimate	Std.Error	*z*-value	Pr (>|*z*|)
Intercept	−1·304	0·315	−4·135	*P* < 0·0001
Age-27	0·042	0·009	4·456	*P* < 0·0001
Wounding (very light and light)	1·637	0·612	2·675	0·007
Wounding (moderate and severe)	4·737	1·173	4·039	*P* < 0·0001
Mature	0·957	0·340	2·816	0·005
(Age-27) × Wounding (very light and light)	−0·032	0·012	−2·729	0·006
(Age-27) × Wounding (moderate and severe)	−0·056	0·013	−4·236	*P* < 0·0001

An estimate of the probability of infection at weaning is given by the intercept term in the final model. Thus, the youngest animal trapped (27-days-old), and so likely to have just left the nest, without wounding and sexually immature, had an estimated probability of infection of 0·21 (95% confidence interval: 0·13–0·33).

### Survival analysis

The final survival model included wounding, sexual maturity, sex and an interaction term between wounding and sexual maturity (Table 3). Having wounds, being sexually mature and being female increased the risk of infection, but beyond these individual effects, being both wounded and sexually mature decreased the risk of infection. The estimate of the shape parameter *κ* was 0·81 (95% confidence interval 0·52–1·28). Hence, there was no significant change in the risk of infection as the rats aged.

**Table 3.** Summary of final survival model fit (AIC = 386)

	Estimate	Std. Error	*z* value	*P* (>|*z*|)
(Intercept)	4·935	0·366	13·50	*P* < 0·0001
Wounded	−1·228	0·553	2·219	0·026
Mature	−1·232	0·474	2·596	0·009
Sex (female)	−0·362	0·184	1·973	0·048
Wounded × Mature	1·123	0·532	2·112	0·035

**Fig. 3.**
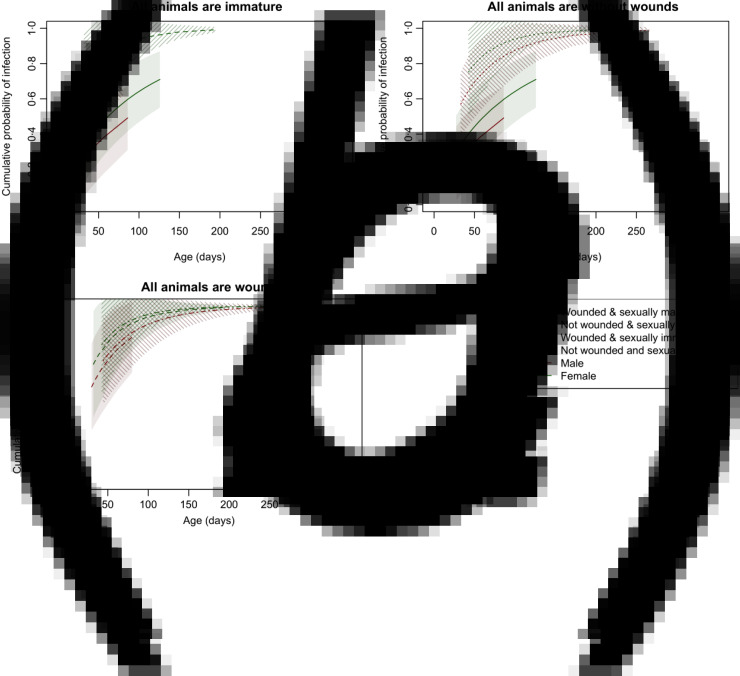
The cumulative distribution function of the Weibull distribution with parameters estimated from the survival model and 95% confidence intervals with standard errors calculated using the delta method illustrating the risk of infection with increasing age when (*a*) all animals are immature, (*b*) all animals are without wounds and (*c*) all animals are wounded.

These effects are more clearly seen in Figure 3, which shows the cumulative distribution function of the Weibull distribution with parameters estimated from the survival model and standard errors calculated using the delta method (see Supplementary Materials for more detail). As well as females having a consistently higher risk of infection than males, wounding clearly increased the risk of infection among immature animals (Fig. 3*a*); sexual maturity increased the risk of infection among those without wounds (Fig. 3*b*); but for those with wounds, there was no significant difference in risk between mature and immature animals (Fig. 3*c*).

## DISCUSSION

Factors related to increased risk of infection do not directly translate to the reality of transmission risk. We used two model frameworks to infer risk of infection with different underlying assumptions of risk. The GLM framework aids us in identifying risk factors for infection and the survival framework assumes that animals may be at risk of future infection, which is a more appropriate model framework for our data, as the captured rats may be infected in the future. The results of our two models lead us to conclude that we have obtained evidence of animals leaving the nest with infection but that the risk of infection also occurs via the environment. However, we did not find that direct transmission, via sexual contact or other routes, occurs at an epidemiologically significant rate.

Direct evidence of different transmission routes occurring successfully and significantly in the field is more informative than experimental approaches, which, at best, can only represent the potential for transmission. Additionally, laboratory studies of rats cannot simultaneously investigate all the variables and interaction effects examined in the present study. For example, the acquisition of wounds in wild rat populations and its interactions with other variables could not be investigated in a laboratory setting. For leptospirosis, as for other zoonoses, control of the primary reservoir can reduce the risk of zoonotic pathogen transmission to humans [32]. Key to controlling infection in a reservoir host is an understanding the dynamics of that infection. An understanding of transmission is key to understanding the dynamics of any infection.

Previous studies have used the FOI to understand how transmission occurs in wild populations [19, 20]. The notable difference in our study is that changes in risk were identified based on demographic variables instead of specified hazard functions (piece-wise or step functions, for example). In wildlife systems, there is not a distinct time threshold for when animals reach different phases of their life cycle. For example, the onset of sexual maturity for rats has been found to range between 45 and 95 days for males, and between 45 and 75 days for females [15] and so it would be inappropriate to use a step function to model change in risk for sexual maturity. Non-linear functions of hazard can be specified based on *priori* assumptions about changes in risks, but by allowing covariates to create this change in risk the variation of demographic processes in wild animals can be accounted for. General methods for utilising age-prevalence data are in continuing development. For example, recent developments include a framework to combine age-prevalence data with individual level antibody response data [33] and a framework to estimate individual time since infection using multiple data sources [34].

One hypothesised transmission route of leptospire infection is the vertical transmission. As rats confined to the nest were never sampled, it is impossible to distinguish true vertical (in utero) from pseudo-vertical transmission (e.g. suckling), or from transmission from mother to pups in the burrow. Juvenile rats have been documented to leave the natal burrow at 25 days of age, although initial movements are limited to those close to the natal burrow space [15]. Milk transfer/production by mothers has been estimated to cease by 27 and 30 days postpartum, respectively, corresponding to the onset of free-roaming behaviour [35, 36]. Hence a 27-day-old animal can be taken to be the one that has not been exposed to any of the other transmission routes and its predicted probability of infection of 0·21 (95% confidence interval: 0·13–0·33) and relatively narrow confidence interval, suggest strongly that a significant proportion of animals leave the nest infected. Among the possible mechanisms behind this, pathogenic leptospires are present in the milk and breast tissue of chronically infected lactating females [14], but this does not necessarily indicate that transmission to suckling animals occurs at epidemiologically significant rates. Other studies have reported the isolation of leptospires from foetal Norway rats, so this potential transmission route requires additional study [37].

Our parsing of the risk of rat infection by age, sexual maturity, sex and wounding provides clues as to the relative roles of direct and environmental transmission. Our finding that rats with wounds have a significantly increased risk of infection confirms previous studies carried out in Salvador and Vancouver, Canada [12, 16]. This may be true direct transmission (via biting for example), or an increased risk created by a different behaviour of those animals most likely to be wounded, or an increased risk of transmission from exposure, directly through those wounds, to leptospires in the environment. With regard to biting (mechanical transmission of leptospires contaminating the buccal cavity) Norway rats spend a considerable proportion of their time grooming (~40% for males [38]) and as the average infected rat sheds 5·9 × 10^6^ leptospires per ml of urine [13] there is potential for contamination of saliva with leptospires during urogenital grooming that can subsequently be transmitted by bite. There have been reports of humans acquiring leptospirosis following rat bite [39], which gives some support to this theory. However, our attempts to detect leptospires in the saliva of infected Norway rats found only a minority were positive (7/18) and those that were had concentrations two orders of magnitude less than infective doses used in experimental infection (Zhan *et al.*, unpublished observations). Thus, given this low concentration and the short period of contact between saliva and an open wound in the act of biting, we consider epidemiologically significant transmission by this route very unlikely. It is far more plausible that wounds are a favourable entry port for environmental contamination, where contact with water and soil (where concentrations are similar to that in saliva [13]) is repeated and longer-lasting than biting. We did not see an effect of wounding on mature rats. This may be because most animals are infected by adulthood and so wounding is no longer a risk factor. However, we would expect a difference in risk by wounding level at sexual maturity if older higher ranking animals were less likely to be wounded [15] and hence infected.

In both analyses, sexually mature animals were, overall, more likely to be infected. This could reflect an increased risk of having acquired infection by non-sexual routes, such as a higher cumulative exposure to the environment, or a separate transmission route such as sexual transmission. However, we did not observe a significant change in risk with the onset of sexual maturity in the plots of cumulative risk of infection, after accounting for age and wounding, which would have been an indication of sexual transmission. Among unwounded animals, those that were sexually mature were more likely to be infected but they were also older. Sexual transmission may occur, therefore, and unpublished evidence of leptospires in semen (Zhan *et al.*, unpublished observations) supports this, but, we propose, not at an epidemiologically significant rate.

Finally, the cumulative distribution plots all showed that females had a higher risk of infection than males, but only earlier in life. A previous study of leptospiral infection in Salvadoran rats also found evidence that female rats were infected at higher rates as juveniles than males [12]. Given that there were no interactions between sex and the other variables in the model, there is no evidence to suggest that the additional risk to females is from a one-way sexual transmission route or a differential effect of wounding on the sexes. Instead, the additional risk for females may come from some behavioural or physiological difference between the sexes that is apparent from the early life stages.

Humans most frequently acquire *Leptospira* infection from contact with the environment contaminated with animal urine. To reduce the level of infection in the environment in Salvador, we must target the rodents. Our results on the occurrence of multiple transmission routes can inform which sub-populations of rodents should be targeted. Given the likelihood of animals leaving the nest with infection, we can conclude that targeting nests and burrows will help reduce infection in the environment.

It should be noted that our observations in both the prevalence and survival model are strongly dependent on the imputed age. We used the same growth curve to impute ages for male and female rats based on the growth of male captive-reared wild Norway rats [15]. In the study described in [15], females were on average lighter than males. Female rats were not substantially lighter than male rats in this study, hence using one growth curve for males and females in our study was not an inappropriate choice. However, it has been found than Norway rats grow quicker in urban settings [40]. Eye lens weight is a good predictor of age for Norway rats regardless of location differences [40]. Our inferences are subject to change with different growth rate parameters, but without eye lens samples we had to rely on published growth curves.

The weight of pregnant females was adjusted despite finding a none significant difference in the weights of pregnant and non-pregnant sexually mature females, as pregnant rats would be expected to be heavier than non-pregnant females of the same age. However, omitting the pregnancy adjustment had no material effect on our conclusions.

Using the two modelling approaches, we have inferred evidence of transmission routes based on statistical associations and the data available to us. The weight-prevalence data are from rodents trapped in Salvador, which may give a biased snapshot of the population. In addition, we have dealt with factors that are closely related such as the onset of sexual maturity and the age of the animal. We have attempted to control for these dependencies by testing interactions of the dependent factors, though some confounding effects could exist in our results. Despite the caveats of using statistical results to infer reality, we believe that using two modelling approaches with different underlying assumptions we have been able to identify which transmission routes are occurring in the Salvador rodent population.

This study has illustrated novel methods of identifying evidence of multiple transmission routes of leptospirosis from prevalence data obtained from the field. The survival model extends the standard practice of analysing prevalence data in an epidemiological setting by considering risk over time. The combination of potential vertical, direct and environmental routes is shared with other species and applicable to other zoonotic pathogens. In the present case, we found support for significant risk of infection within the natal burrow – in effect, vertical transmission – and environmental transmission. Beyond that, while direct transmission, either sexually or in the act of biting, is biologically possible, evidence for this being epidemiologically significant was weak. These observations, obtained using data of transmission in the wild, can usefully inform a tractable mathematical model of Norway rat leptospirosis.

## Supplementary material

For supplementary material accompanying this paper visit https://doi.org/10.1017/S0950268817002539. click here to view supplementary material

## References

[1] Lloyd-Smith JO, Epidemic dynamics at the human-animal interface. Science 2009; 326: 1362–1367. 19965751 10.1126/science.1177345 PMC3891603

[2] Ko AI, Goarant C, Picardeau M. *Leptospira*: the Dawn of the molecular genetics era for an emerging zoonotic pathogen. Nature Reviews Microbiology 2009; 7: 736–747. 19756012 10.1038/nrmicro2208 PMC3384523

[3] Shaked Y, Leptospirosis in pregnancy and its effect on the fetus: case report and review. Clinical Infectious Diseases 1993; 17: 241–243. 8399874 10.1093/clinids/17.2.241

[4] Bolin CA, Koellner P. Human-to-human transmission of *Leptospira* interrogans by milk. Journal of Infectious Diseases 1988; 158: 246–227. 3392418 10.1093/infdis/158.1.246

[5] Bharti AR, Reviews leptospirosis: a zoonotic disease of global importance. The Lancet 2003; 3: 757–771. 14652202 10.1016/s1473-3099(03)00830-2

[6] Adler B, de la Peña Moctezuma A. *Leptospira* and leptospirosis. Veterinary Microbiology 2010; 140: 287–296. 19345023 10.1016/j.vetmic.2009.03.012

[7] Riley LW, Slum health: diseases of neglected populations. BMC International Health and Human Rights 2007; 7: 2. 17343758 10.1186/1472-698X-7-2 PMC1829399

[8] Ko AI, Urban epidemic of severe leptospirosis in Brazil. The Lancet 1999; 354: 820–825. 10.1016/s0140-6736(99)80012-9 10485724

[9] Reis RB, Impact of environment and social gradient on *Leptospira* infection in urban slums. PLoS Neglected Tropical Diseases 2008; 2: e228. 18431445 10.1371/journal.pntd.0000228 PMC2292260

[10] Felzemburgh RDM, Prospective study of leptospirosis transmission in an urban slum community: role of poor environment in repeated exposures to the leptospira agent. PLoS Neglected Tropical Diseases 2014; 8: e2927. 24875389 10.1371/journal.pntd.0002927 PMC4038618

[11] Ganoza CA, Determining risk for severe leptospirosis by molecular analysis of environmental surface waters for pathogenic *Leptospira*. PLoS Medicine 2006; 3: e308. 16933963 10.1371/journal.pmed.0030308 PMC1551915

[12] Costa F, Infections *by Leptospira interrogans*, Seoul virus, and *Bartonella* spp. among Norway rats (*Rattus norvegicus*) from the urban slum environment in Brazil. Vector Borne and Zoonotic Diseases 2014; 14: 33–40. 24359425 10.1089/vbz.2013.1378 PMC3880909

[13] Costa F, Patterns in *Leptospira* shedding in Norway rats (*Rattus norvegicus*) from Brazilian slum communities at high risk of disease transmission. PLOS Neglected Tropical Diseases 2015; 9: e0003819. 26047009 10.1371/journal.pntd.0003819 PMC4457861

[14] De Oliveira D, *Leptospira* in breast tissue and milk of urban Norway rats (*Rattus norvegicus*). Epidemiology and Infection 2016; 144: 1–10. 27019024 10.1017/S0950268816000637 PMC5437553

[15] Calhoun JB. The Ecology and Sociology of the Norway rat (US Public Health Service Publication no. 1008). Washington, DC: US Government Printing Office, 1962.

[16] Himsworth CG, Ecology of *Leptospira interrogans* in Norway rats (*Rattus norvegicus*) in an inner-city neighborhood of Vancouver, Canada. PLoS Neglected Tropical Diseases 2013; 7: e2270. 23818996 10.1371/journal.pntd.0002270 PMC3688548

[17] Hinson ER, Wounding: the primary mode of Seoul virus transmission among male Norway rats. American Journal of Tropical Medicine and Hygiene 2004; 70: 310–317. 15031523

[18] Caley P, Ramsey D. Estimating disease transmission in wildlife, with emphasis on leptospirosis and bovine tuberculosis in possums, and effects of fertility control. Journal of Applied Ecology 2001; 38: 1362–1370.

[19] Long GH, Identifying the age cohort responsible for transmission in a natural outbreak of *Bordetella bronchiseptica*. PLoS Pathogens 2010; 6: e1001224. 21187891 10.1371/journal.ppat.1001224 PMC3002977

[20] Caley P, Hone J. Disease transmission between and within species, and the implications for disease control. Journal of Applied Ecology 2004; 41: 94–104.

[21] McCallum H, Barlow N, Hone J. How should pathogen transmission be modelled? Trends in Ecology & Evolution 2001; 16: 295–300. 11369107 10.1016/s0169-5347(01)02144-9

[22] Heisey DM, Joly DO, Messier F. The fitting of general force of infection models to wildlife disease prevalence data. Ecology 2006; 87: 2356–2365. 16995636 10.1890/0012-9658(2006)87[2356:tfogfm]2.0.co;2

[23] Panti-May JA, A two-year ecological study of Norway rats (*Rattus norvegicus*) in a Brazilian urban slum. PLoS ONE 2016; 11: e0152511. 27015422 10.1371/journal.pone.0152511 PMC4807843

[24] Glass GE, Association of intraspecific wounding with hantaviral infection in wild rats (*Rattus norvegicus*). Epidemiology and Infection 1988; 101: 459–472. 3141203 10.1017/s0950268800054418 PMC2249393

[25] Iskjaer CC, Slade N, Childs J. Body mass as a measure of body size in small mammals. Journal of Mammalogy 1989; 70: 662–667.

[26] Glass G, Korch G, Childs J. Seasonal and habitat differences in growth rates of wild *Rattus norvegicus*. Journal of Mammalogy 1988; 69: 587–592.

[27] Burthe SJ, Individual growth rates in natural field vole, *Microtus agrestis*, populations exhibiting cyclic population dynamics. Oecologia 2010; 162: 653–661. 19916066 10.1007/s00442-009-1495-6

[28] R Core Team. Statistical, R: A Language and Environment for Computing. Vienna, Austria: R Foundation for Statistical Computing, 2017.

[29] Kosmidis I. brglm: Bias reduction in binary-response Generalized Linear Models. 2007 (http://www.ucl.ac.uk/~ucakiko/software.html).

[30] Firth D. Bias reduction of maximum likelihood. Biometrika 1993; 80: 27–38.

[31] Ellis WA. Animal leptospirosis. In: Adler B, ed. Leptospira *and Leptospirosis*. Berlin: Springer, 2015, pp. 99–137.

[32] Childs JE, Animal-based national surveillance for zoonotic disease: quality, limitations, and implications of a model system for monitoring rabies. Preventive Veterinary Medicine 2007; 78: 246–261. 17129622 10.1016/j.prevetmed.2006.10.014 PMC7114326

[33] Pepin KM, Inferring infection hazard in wildlife populations by linking data across individual and population scales. Ecology Letters 2017; 20: 275: 292. 28090753 10.1111/ele.12732 PMC7163542

[34] Borremans B, Estimating time of infection using prior serological and individual information can greatly improve incidence estimation of human and wildlife infections. PLoS Computational Biology 2016; 12: e1004882. 27177244 10.1371/journal.pcbi.1004882 PMC4866769

[35] Galef BG. The ecology of weaning. In: Gubernick DJ and Klopfer PH., eds. Parental Care in Mammals. New York: Plenum Press, 1981, pp. 211–241.

[36] Thiels E, Cramer CP, Alberts JR. Behavioral interactions rather than milk availability determine decline in milk intake of weanling rats. Physiology & Behavior 1988; 42: 507–515. 3413227 10.1016/0031-9384(88)90152-7

[37] Lahiri MN. On the foetal infection by *L. Icterohaemorrhagiae* in a rat. The Indian Journal of Medical Research 1941; 29: 685–688.

[38] Bolles RC. Grooming behavior in the rat. Journal of Comparative and Physiological Psychology 1960; 53: 306–310. 13802322 10.1037/h0045421

[39] Roczek A, Severe course of rat bite-associated Weil's disease in a patient diagnosed with a new *Leptospira*-specific real-time quantitative LUX-PCR. Journal of Medical Microbiology 2008; 57: 658–663. 18436602 10.1099/jmm.0.47677-0

[40] Feng AYT, Himsworth CG. The secret life of the city rat: a review of the ecology of urban Norway and black rats (*Rattus Norvegicus* and *Rattus Rattus*). Urban Ecosystems 2014; 17: 149–162.

