# Supplementary material for: Evidence of multiple intraspecific transmission routes for Leptospira acquisition in Norway rats (Rattus norvegicus)
Source: Epidemiol Infect. 2017 Dec;145(16):3438–48. doi: 10.1017/S0950268817002539 (PMC6252042; doi:10.1017/S0950268817002539)
Supplement: Supplementary file 1 [file S0950268817002539sup001.docx]

Epidemiology and Infection

Evidence of multiple intraspecific transmission routes for *Leptospira* acquisition in Norway rats (*Rattus norvegicus)*

A. Minter, P.J. Diggle, F. Costa, J. Childs, A. I. Ko, M. Begon

Supplementary Material

S1. Survival model

If the probability of not yet being infected is modelled using the cumulative distribution function from the Weibull distribution, then,

$$P\left( Y_{i}=0 \right)=1-F\left( t_{i} \right)$$

$$\log\left( P\left( Y_{i}=0 \right) \right)=\log\left( \exp(-\left( \frac{t_{i}}{\phi} \right)^{\kappa} \right)$$

$$-\log\left( P\left( Y_{i}=0 \right) \right)=\left( \frac{t_{i}}{\phi} \right)^{\kappa}$$

$$\log\left( -\log\left( P\left( Y_{i}=0 \right) \right) \right)=\kappa\log\left( t_{i} \right)-\kappa\log\left( \phi\right)$$

if we chose to model the scale parameter as log linear, then $\log\left( \phi\right)=X\boldsymbol{\beta}$ and so,

$\log\left( -\log\left( P\left( Y_{i}=0 \right) \right) \right)=\kappa\log\left( t_{i} \right)-\kappa X\boldsymbol{\beta}$.

Then we can estimate coefficients $\boldsymbol{\beta}$ by maximising the likelihood function,

$$L\left( \beta|x_{i} \right)=\prod_{i=1}^{n} p_{i}^{y_{i}}{(1-p_{i})}^{1-y_{i}}$$

where $p_{i}$is the probability of already being infected, with $p_{i}=1-\exp\left( -\exp\left( \kappa\log\left( t_{i} \right)-\kappa X\boldsymbol{\beta} \right) \right)$.

S2. Delta method

The delta method (Oehlert 1992) was used to find the standard errors of the Weibull cumulative distribution function (cdf) $F\left( t,X;\kappa,\boldsymbol{\beta} \right)=1-\exp(-{(t/\phi)}^{\kappa})$. The variance matrix of the Weibull cdf is,

$Var\left( F\left( t,X;\kappa,\boldsymbol{\beta} \right) \right)\approx\nabla{F\left( t,X;\kappa,\boldsymbol{\beta} \right)}^{T}.Cov\left( X \right).\nabla F(t,X;\kappa,\boldsymbol{\beta})$.

Where $\nabla F(t,X;\kappa,\boldsymbol{\beta})$ is the vector of partial derivatives of $F(t,X;\kappa,\boldsymbol{\beta})$ with respect to the model parameters and $Cov\left( t,X \right)$ is the covariance matrix. The covariance matrix was estimated by numerical approximation of the hessian matrix.

Rewrite the cdf as $F\left( t,X;\kappa,\boldsymbol{\beta} \right)=1-\exp\left( -\left( t\exp\left( -\eta\right) \right)^{\kappa} \right)$ with $\eta=X\boldsymbol{\beta}$. In the final model $\eta=\beta_{0}+\beta_{1}x_{1}+\beta_{2}x_{2}+\beta_{3}x_{3}+\beta_{4}x_{1}x_{2}$. As the shape parameter is strictly positive, we specify the shape parameter as $\kappa=exp(\kappa^{*})$, hence our covariance matrix is for the parameter $\kappa^{*}$. We must calculate the standard errors of$F\left( t,X;\kappa,\boldsymbol{\beta} \right)$ with respect to $\kappa^{*}$. The partial derivatives were,

$\frac{\partial F\left( t,X;\kappa,\boldsymbol{\beta} \right)}{\partial\kappa^{*}}=\log\lambda.\lambda^{exp(\kappa^{*})}\exp\left( -\lambda^{exp(\kappa^{*})} \right)exp(\kappa^{*})$ where $\lambda=t\exp\left( -\eta\right)$

$$\frac{\partial F\left( t,X;\kappa,\boldsymbol{\beta} \right)}{\partial\beta_{0}}=\kappa t^{\kappa}\exp\left( -\eta\kappa\right)\exp\left( -t^{\kappa} \exp\left( -\eta\kappa\right) \right)$$

$$\frac{\partial F\left( t,X;\kappa,\boldsymbol{\beta} \right)}{\partial\beta_{1}}=\kappa x_{1}t^{\kappa}\exp\left( -\eta\kappa\right)\exp\left( -t^{\kappa} \exp\left( -\eta\kappa\right) \right)$$

$$\frac{\partial F\left( t,X;\kappa,\boldsymbol{\beta} \right)}{\partial\beta_{2}}=\kappa x_{2}t^{\kappa}\exp\left( -\eta\kappa\right)\exp\left( -t^{\kappa} \exp\left( -\eta\kappa\right) \right)$$

$$\frac{\partial F\left( t,X;\kappa,\boldsymbol{\beta} \right)}{\partial\beta_{3}}=\kappa x_{3}t^{\kappa}\exp\left( -\eta\kappa\right)\exp\left( -t^{\kappa} \exp\left( -\eta\kappa\right) \right)$$

$\frac{\partial F\left( t,X;\kappa,\boldsymbol{\beta} \right)}{\partial\beta_{4}}=\kappa x_{4}t^{\kappa}\exp\left( -\eta\kappa\right)\exp\left( -t^{\kappa} \exp\left( -\eta\kappa\right) \right)$.
