# Supplementary material for: Evidence of multiple intraspecific transmission routes for Leptospira acquisition in Norway rats (Rattus norvegicus)
Source: Epidemiol Infect. Author manuscript; Available in PMC 2018 Nov 24. (PMC6252042; doi:10.1017/S0950268817002539)
Supplement: Supplementary file 1 [file NIHMS994423-supplement-Supplemental_Material.pdf]

Epidemiology and Infection

Evidence of multiple intraspecific transmission routes for *Leptospira* acquisition in Norway rats (*Rattus norvegicus)*

A. Minter, P.J. Diggle, F. Costa, J. Childs, A. I. Ko, M. Begon

Supplementary Material

S1. Survival model

If the probability of not yet being infected is modelled using the cumulative distribution function from the Weibull distribution, then,

$$P(Y_i = 0) = 1 - F(t_i)$$

$$\log\big(P(Y_i = 0)\big) = \log\left(\exp(-\left(\frac{t_i}{\phi}\right)^{\kappa}\right)$$

$$-\log\big(P(Y_i = 0)\big) = \left(\frac{t_i}{\phi}\right)^{\kappa}$$

$$\log\big(-log\big(P(Y_i = 0)\big)\big) = \kappa\log(t_i) - \kappa\log(\phi)$$

if we chose to model the scale parameter as log linear, then $\log(\phi) = X\boldsymbol{\beta}$ and so,

$$\log\big(-log\big(P(Y_i = 0)\big)\big) = \kappa\log(t_i) - \kappa X\boldsymbol{\beta}.$$

Then we can estimate coefficients $\boldsymbol{\beta}$ by maximising the likelihood function,

$$L(\beta|x_i) = \prod_{i=1}^{n} p_i^{y_i}(1 - p_i)^{1-y_i}$$

where $p_i$is the probability of already being infected, with $p_i = 1 - \exp(-exp(\kappa\, log(t_i) - \kappa X\boldsymbol{\beta}))$.

S2. Delta method

The delta method (Oehlert 1992) was used to find the standard errors of the Weibull cumulative distribution function (cdf) $F(t, X; \kappa, \boldsymbol{\beta}) = 1 - \exp(-(t/\phi)^{\kappa})$. The variance matrix of the Weibull cdf is,

$$Var\big(F(t, X; \kappa, \boldsymbol{\beta})\big) \approx \nabla F(t, X; \kappa, \boldsymbol{\beta})^{T}.\, Cov(X).\, \nabla F(t, X; \kappa, \boldsymbol{\beta}).$$

Where $\nabla F(t, X; \kappa, \boldsymbol{\beta})$ is the vector of partial derivatives of $F(t, X; \kappa, \boldsymbol{\beta})$ with respect to the model parameters and $Cov(t, X)$ is the covariance matrix. The covariance matrix was estimated by numerical approximation of the hessian matrix.

Rewrite the cdf as $F(t, X; \kappa, \boldsymbol{\beta}) = 1 - \exp\Big(-\big(t\exp(-\eta)\big)^{\kappa}\Big)$ with $\eta = X\boldsymbol{\beta}$. In the final model $\eta = \beta_0 + \beta_1 x_1 + \beta_2 x_2 + \beta_3 x_3 + \beta_4 x_1 x_2$. As the shape parameter is strictly positive, we specify the shape parameter as $\kappa = \exp(\kappa^*)$, hence our covariance matrix is for the parameter κ^*. We must calculate the standard errors of $F(t, X; \kappa, \boldsymbol{\beta})$ with respect to κ^*. The partial derivatives were,

$$\frac{\partial F(t, X; \kappa, \boldsymbol{\beta})}{\partial \kappa^*} = \log \lambda \,.\, \lambda^{\exp(\kappa^*)} \exp\big(-\lambda^{\exp(\kappa^*)}\big) \exp(\kappa^*) \quad \text{where} \quad \lambda = t\exp(-\eta)$$

$$\frac{\partial F(t, X; \kappa, \boldsymbol{\beta})}{\partial \beta_0} = \kappa\, t^{\kappa} \exp(-\eta\kappa) \exp\big(-t^{\kappa} \exp(-\eta\kappa)\big)$$

$$\frac{\partial F(t, X; \kappa, \boldsymbol{\beta})}{\partial \beta_1} = \kappa x_1\, t^{\kappa} \exp(-\eta\kappa) \exp\big(-t^{\kappa} \exp(-\eta\kappa)\big)$$

$$\frac{\partial F(t, X; \kappa, \boldsymbol{\beta})}{\partial \beta_2} = \kappa x_2\, t^{\kappa} \exp(-\eta\kappa) \exp\big(-t^{\kappa} \exp(-\eta\kappa)\big)$$

$$\frac{\partial F(t, X; \kappa, \boldsymbol{\beta})}{\partial \beta_3} = \kappa x_3\, t^{\kappa} \exp(-\eta\kappa) \exp\big(-t^{\kappa} \exp(-\eta\kappa)\big)$$

$$\frac{\partial F(t, X; \kappa, \boldsymbol{\beta})}{\partial \beta_4} = \kappa x_4\, t^{\kappa} \exp(-\eta\kappa) \exp\big(-t^{\kappa} \exp(-\eta\kappa)\big).$$
